# Health Effects of Bee Products: A Comprehensive Review

**DOI:** 10.1002/fsn3.71165

**Published:** 2026-02-03

**Authors:** Nevin Sanlier, Elif Yildiz Kaya, Ikbal Irem Yucel

**Affiliations:** ^1^ Department of Nutrition and Dietetics, School of Health Sciences Ankara Medipol University Altındağ Ankara Turkey; ^2^ Department of Nutrition and Dietetics Institute of Health Sciences, Ankara Medipol University Altındağ Ankara Turkey

**Keywords:** apitherapy, bee pollen, bee products, honey, propolis

## Abstract

Honey, bee pollen, propolis, bee bread, royal jelly, bee venom, beeswax, and apilarnil are among the bee‐derived products that may serve health‐related purposes, as they exhibit various biological activities such as antibacterial, antiviral, antifungal, antioxidant, anti‐inflammatory, antitumor, vasodilatory, and blood pressure‐lowering effects. In this review, the possible health effects and action mechanisms of bee products frequently used in apitherapy were examined. The therapeutic potential of bee‐derived products is attributed to their content of amino acids, fatty acids, carbohydrates, vitamins, minerals, flavonoids, polyphenols, and various other bioactive components that contribute to immune system support. In addition, it is known that these products have anti‐inflammatory, antimicrobial, and immunomodulatory effects, thanks to their bioactive component content, and have a positive effect on cancer, diabetes, and cardiovascular and neurological diseases caused by oxidative stress. The lack of standardization of bee products hinders the clarity of relevant studies. To comprehensively clarify the health impacts of bee‐derived products, further research employing standardized preparations is required. Future research involving clinical trials of bee‐derived products with varying dosages and durations may help elucidate their potential link to human health. Additionally, epidemiological and clinical studies are needed on the usefulness of bee products to make generalizations.

## Graphical abstract

Nutritional composition of bee products and some health effects.

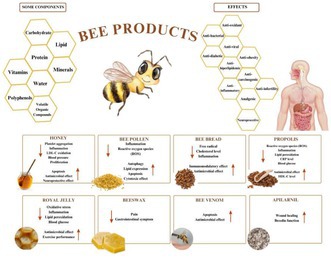

**Abbreviations**
AD: Alzhemiers disease
ARC: artepillin C
CAPE: caffeic acid phenyl ester
CRP: C‐reactive protein
DBH: drone brode homogenate
EFSA: European Food Safety Authority
HDL‐C: high density lipoprotein cholesterol
HMF: hydroxymethylfurfural
IL‐6: interleukin‐6
LDL‐C: low density lipoprotein cholesterol
MetS: metabolic syndrome
NAFLD: non‐alcoholic fatty liver
PAPP: pectic bee pollen polysaccharides
PLA2: phospholipase A2
ROS: reactive oxygen species
T2DM: type 2 diabetes mellitus
TC: total cholesterol
TNF‐α: tumor necrosis factor‐α
USDA: United States Department of Agriculture
WHO: World Health Organization

## 1 Introduction

For over 5500 years, bee‐derived products have been recognized for both their nutritional value and their healing properties. In numerous ancient civilizations, honey and other bee‐derived products served both dietary and medicinal purposes (Frunze et al. 2021). These products are also mentioned in religious texts, with honey described as a source of healing (Nader et al. 2021). Apitherapy, in which bee‐derived products such as honey, bee pollen, propolis, bee bread (perga), royal jelly, bee venom (apitoxin), and beeswax are used to protect health and prevent or manage diseases, is within the scope of traditional and complementary medicine practices (Ali and Kunugi 2020a).

Owing to their content of flavonoids, polyphenols, and various other bioactive compounds, bee‐derived products draw interest as adjuncts or alternatives to modern medical approaches in both the prevention and treatment of numerous diseases (Dumitru et al. 2022). Bee products have many antimicrobial, antioxidant, anticarcinogenic, antidiabetic, antimutagenic, antiviral, antifungal, immunomodulatory, anti‐inflammatory, neuroprotective, and immune system‐supporting effects arising from their amino acids, fatty acids, carbohydrates, vitamins, minerals, flavonoids, polyphenols, and various additional bioactive constituents. They are reported to have significant therapeutic potential for disease prevention and management (Durazzo et al. 2021). The chemical profile of bee‐derived products can differ according to the plants surrounding the hive, the geographical region, and climatic characteristics (Darwish et al. 2022). For these reasons, bee products are generally not used in modern medicine because of the lack of standardization, despite their beneficial effects (Ali and Kunugi 2020a).

In this review, beekeeping products frequently used in apitherapy, their possible health effects, and their mechanisms of action are examined.

## 2 Methodology

Using the PubMed, Web of Science, and Google Scholar databases, a comprehensive literature search of original articles published in recent years was conducted using the following keywords: ‘bee products’, ‘honey’, ‘bee pollen’, ‘propolis’, ‘bee bread’, ‘royal jelly’, ‘bee venom’, ‘beeswax’, ‘apilarnil’, and ‘bee products and health’. No additional restrictions were imposed other than the selected search terms.

The study included original research, systematic review, meta‐analysis, review, letter to the editor, in vitro, and animal or clinical human studies with the full text format in English. The studies were accessed using the databases and keywords specified in the “Methodology” section. Articles written in non‐English languages or published as pre‐prints were excluded from the screening process. Studies between the years 2015 and 2025 were included in the review.

## 3 Bee Products

### 3.1 Honey

Honey refers to a natural product obtained when honey bees ( *Apis mellifera* ) gather nectar from plant blossoms or collect plant secretions (Pereira et al. 2023). After flower nectar or plant secretions are collected by honey bees, their compositions change in the bodies of the bees; they are combined with other special substances, and they mature after being stored in honeycomb cells. When pollen and nectar gathered by honey bees originate from plants, the product is classified as “flower honey”. In contrast, if the bees utilize secretions from plants or from insects inhabiting them, the outcome is referred to as “secretion honey” (Hossain et al. 2022). Flower honey is also classified as monofloral honey or multifloral honey according to the pollen and nectar diversity. If the pollen and nectar ratio of a particular plant in honey is over 45% or if the pollen and nectar source is a single plant, the honey is categorized as monofloral, and if the pollen and nectar are sourced from many plants, the honey is multifloral. Cotton, sunflower, and chestnut honey are examples of monofloral honey; plateau honey is an example of multifloral honey; and pine honey is an example of secretion honey (Machado et al. 2020).

Honey's mineral, water, and sugar composition, along with physical and chemical characteristics including pH level and enzyme activity, can differ according to the origin of the pollen (Santos et al. 2021). The main component of honey is carbohydrates (Seraglio et al. 2021). The sugar in honey comprises 70% fructose and glucose and 10% maltose, sucrose, trehalose, isomaltose, and others. Fructose is most prevalent among the sugars in honey (Kozłowicz et al. 2020), and the amount of protein is quite low. However, amino acid diversity is high. Proline is the predominant amino acid present (Santos et al. 2021). Other amino acids are histidine, aspartic acid, glycine, glutamic acid, glutamine, threonine, tyrosine, alanine, tryptophan, threonine, methionine, lysine, serine, cysteine, asparagine, leucine, and phenylalanine (Kozłowicz et al. 2020). Honey is rich in potassium and also provides a range of minerals, including calcium, iron, magnesium, phosphorus, sodium, zinc, copper, manganese, selenium, and fluorine (USDA 2019). It is also rich in polyphenols in the form of flavonoids and phenolic acid. Honey contains a variety of flavonoids including apigenin, pinocembrin, kaempferol, quercetin, galangin, chrysin, hesperitin as well as phenolic acids such as ellagic, caffeic, p‐coumaric, and ferulic acid, all of which are recognized for their antioxidant activity (Becerril‐Sánchez et al. 2021).

### 3.2 Bee Pollen

The reproductive material occurring in the male organs of flowering plants is called pollen. Bee pollen constitutes dried pollen pellets collected by honey bees. Bee pollen is a beekeeping product primarily composed of flower pollen collected from various plant species. For thousands of years, it has been incorporated into both traditional medicine and human diets for its recognized therapeutic, disease‐preventive, nutritional, and physiological benefits (Conte et al. 2020). Pollen meets the protein, fat, and vitamin needs of honey bees. The pollen collected by bees is made sticky by their saliva, and the pollen subsequently takes the form of a lump (Ranneh et al. 2021). A single bee colony is capable of gathering approximately 50–250 g of pollen daily, which amounts to about 15–40 kg annually (Thakur and Nanda 2020).

In general, pollen contains 7.5%–40% protein, 15%–50% sugar, and very high amounts of starch ranging from 15% to 50% (Thakur and Nanda 2020). It also contains many amino acids, including essential amino acids, and myristic, stearic, palmitic, oleic, α‐linolenic, and linoleic acids together with provitamin A, E, D, C, and B group vitamins (Baky et al. 2023). It also contains minerals such as potassium, calcium, magnesium, chlorine, phosphorus, silicon, iron, and sodium, along with trace amounts of heavy metals including arsenic, cadmium, and lead (Knoll and Cappai 2024). Phenolic acids are bioactive components found at a rate of approximately 0.19% in pollen. The primary phenolic constituents of bee pollen include benzoic acid, phenylacetic acid, and cinnamic acid. Their derivatives, chlorogenic, gallic, caffeic, ferulic, and hydroxycinnamic acids, also strengthen the antioxidant and radical scavenging effects of pollen (Mărgăoan et al. 2019). Flavonoids are the most remarkable phenolic compounds in bee pollen. Luteolin, apigenin, chrysin, quercetin, kaempferol, myricetin, galangin, naringenin, pinocembrin, genistein, anthocyanidins, and catechins are the main flavonoids of bee pollen and are present around 0.25%–1.4% (Rodríguez‐Pólit et al. 2023).

### 3.3 Propolis

Propolis is a resin‐like material produced when honey bees mix secretions gathered from leaves, flower buds, and tree bark crevices with components such as enzymes and beeswax (Olegário et al. 2019). Bees create propolis to protect hives from infection and to prevent other creatures from entering the hive (Forma and Bryś 2021).

The chemical makeup of propolis changes according to the plant species surrounding the hive and the season of collection. The typical composition of propolis is 50% vegetable resin and balsam, 30% beeswax, 10% aromatic oils, 5% pollen, and 5% other ingredients (Kasote et al. 2022). Flavonoids constitute the principal constituents of propolis. There are many phenolic compounds in propolis, including stilbenes and lignans. Over 100 phenolic compounds have been identified in various types of propolis, including quercetin, CAPE, p‐vanillin, p‐coumaric acid, apigenin, caffeic acid, cinnamic acid, pinobanksin 5‐methyl ether, quercetin 7‐methyl ester, trans‐ferulic acid, veratric acid, aldehyde benzoic acid, and cis‐ferulic acid (Dezmirean et al. 2020).

Although propolis has been shown to be beneficial in the treatment of various diseases, its practical use is hindered by the lack of standardization of its chemical composition. Even if propolis samples are collected from the same region, their contents may vary depending on the season. Therefore, the effect mechanisms of propolis must be further clarified with long‐term studies conducted with standard samples (El‐Guendouz et al. 2019).

### 3.4 Bee Bread

Bee pollen serves as the raw material used by bees to produce bee bread. Bee bread is a fermented final product formed from a combination of saliva, pollen, and nectar (Didaras et al. 2021). It is formed as a result of the fermentation of bee pollen with various digestive enzymes and honey (Mărgăoan et al. 2019). Bee bread offers greater nutritional value and higher digestibility compared to pollen. Although the outer layer of pollen cannot be digested in the stomach under normal conditions, the outer layer of the pollen dissolves as a result of pollen fermentation and the substances in the pollen can be absorbed more easily (Bakour et al. 2022). Bees ferment pollen to preserve its high quality and increase the bioavailability of nutrients (Kieliszek et al. 2023).

Bee bread is composed of roughly 20% protein, 3% lipids, 24%–35% carbohydrates, and about 3% minerals and vitamins. It serves as the primary protein source for a bee colony and provides polyunsaturated fatty acids such as omega‐3 (α‐linolenic acid), omega‐6 (linoleic acid), and palmitic acid that cannot be synthesized by the human body. It contains all essential amino acids, polyphenols, sterols, carotenoids, various enzymes, and vitamins C, B, E, and K (Mărgăoan et al. 2019; Mohammad et al. 2021). Its polyphenol profile comprises phenolic acids and flavonoids, including kaempferol, myricetin, luteolin, rosmarinic acid, p‐coumaric acid, caffeic acid, and quercetin (Dranca et al. 2020). Thanks to its bioactive compounds, bee bread possesses antiallergic, anti‐inflammatory, and anticancer properties (Mărgăoan et al. 2019).

Fresh bee pollen contains 21%–30% water, which causes microorganisms to grow and pollen to deteriorate. Because bee bread is fermented and acidic, it is more durable than bee pollen, and drying bee bread under appropriate conditions increases its durability (Mărgăoan et al. 2019; Forma and Bryś 2021).

### 3.5 Royal Jelly

Royal jelly is a thick, acidic, yellowish secretion that serves as the lifelong diet of queen bees and is provided to worker bees during the first 3 days of their lives. It is produced by the hypopharyngeal and mandibular glands of worker bees after pollen and nectar undergo digestion in their digestive tracts (Bagameri et al. 2022). Royal jelly contains fat, protein, carbohydrates, P, Na, K, Ca, Mg, vitamin C, vitamin E, and B group vitamins. Seasonal factors can greatly influence the chemical makeup of royal jelly (Kunugi and Mohammed Ali 2019). Although the values vary in different royal jellies, 60%–70% of royal jelly consists of water, 10%–20% sugar, 11%–18% protein, and 1%–4% 10‐hydroxy‐2‐decenoic acid. The most abundant fatty acids in royal jelly are 10‐hydroxydecanoic acid, 10‐hydroxy‐2‐decenoic acid, and sebacic acid (Kunugi and Mohammed Ali 2019). Royal jelly also contains adenosine, adenosine monophosphate, and adenosine monophosphate N1 oxide. The flavonoid contents of royal jelly include flavanols such as quercetin, kaempferol, galangin, and fisetin; it also has flavanones such as pinocembrin, naringin, hesperidin and flavones such as apigenin, acacetin, chrysin, and luteolin (Peršurić and Pavelić 2021).

### 3.6 Bee Venom

Bee venom is a clear, odorless, acidic fluid produced by a gland in the bee's abdominal cavity and utilized for defense against predators. Upon exposure to air, it dries, crystallizes, and takes on a pale yellow color (Khalil et al. 2021). Bee venom contains melittin, apamin, mast cell degranulation peptide, adolapin, PLA2, hyaluronidase, amino acids, and volatile compounds. Melittin is the main component of bee venom and constitutes 40%–60% of its volume. PLA2 is the main allergen of bee venom and constitutes 10%–12% of its volume (Wehbe et al. 2019).

### 3.7 Beeswax

Beeswax is a complex liquid secreted by the wax glands in the abdomens of young worker bees for constructing honeycombs. When first secreted, it is nearly white, but it develops a deep yellowish hue after contact with honey and pollen (Nong et al. 2023). Beeswax contains hydrocarbons, monoesters, diesters, triesters, hydroxy monoesters, hydroxypolyesters, free acids, acid monoesters, and acid polyesters. One of the products made from beeswax is Abexol (D‐002). Abexol is a natural blend of high–molecular weight alcohols extracted and refined from the wax of *Apis mellifera* (Hosseini et al. 2023).

### 3.8 Apilarnil

Drone brood (drone larvae) is commonly utilized in certain countries worldwide, including Romania, China, Zambia, Senegal, and Ecuador. These drones emerge from the drone cells on the 24th day after the eggs are laid and can be easily identified by their distinct physical characteristics (Jensen et al. 2019; González et al. 2024). DBH, known as apilarnil or less commonly apistimul, is a bee product obtained by collecting drone cells from drone broods after hatching. Apilarnil is the male equivalent of royal jelly and is freeze‐dried after being obtained from male bee broods (Sidor and Dżugan 2020; Abd El‐Wahed et al. 2024). DBH is the origin of male (testosterone) and female (estradiol, progesterone, and prolactin) sex hormones. Estradiol and prolactin are found in the highest amounts, whereas testosterone is the lowest (Sidor and Dżugan 2020).

Apilarnil has many biologically active compounds, including proteins, enzymes, carbohydrates, lipids, nucleic acids, steroids, hormones, flavonoids, vitamins, minerals, and essential amino acids (Ghosh et al. 2020; Sidor et al. 2021). It is abundant in carbohydrates, primarily composed of glucose, trehalose, and glycogen (Ghosh et al. 2020). It provides all essential amino acids and is particularly rich in aspartic acid, glutamic acid, proline, valine, leucine, and lysine. It also exhibits antioxidant activity because of its relatively high cysteine content and sulfhydryl groups (‐SH), which reflect the presence of cysteine (Sawczuk et al. 2019). It contains plant sterols such as campesterol, β‐sitosterol, stigmasterol, and 5‐hydroxysitosterol (Sidor and Dżugan 2020). It is also rich in water‐soluble vitamins, including ascorbic acid, B1, B2, B3, folic acid, B5, B6, the B12 group, choline, biotin, and inositol, while containing only low amounts of lipophilic vitamins (Abd El‐Wahed et al. 2024). The chemical composition of DBH resembles that of royal jelly, but it contains less protein and total hydrocarbons, along with a higher water content (Sawczuk et al. 2019). In addition, enzymatic activities and polyphenolic profiles constitute the most significant differences between the two products. Unlike royal jelly, apilarnil contains diastase, α‐glucosidase enzymes, ellagic acid, and ferulic acid (Ghosh et al. 2020).

## 4 Health Effects of Bee Products

### 4.1 Antioxidant and Anti‐Inflammatory Effects

A study examining the incorporation of bee pollen into gluten‐free bread formulations reported notable enhancements in polyphenol bioaccessibility, carotenoid levels, and antioxidant capacity in the enriched bread samples (Conte et al. 2020). Regarding antioxidant potential, formulations incorporating varying ratios of bee pollen and Aronia exhibited strong antioxidative properties (Tirla et al. 2023). However, there are not enough studies to support its use in athletes. It was determined that bee pollen supplementation had hepatoprotective effects compared to whey protein, and rats supplemented with bee pollen had lower levels of antioxidant activities than those that received whey protein (Jarosz et al. 2022).

Propolis has many antioxidant, anti‐inflammatory, anticarcinogenic, immunostimulating, antiviral, antimicrobial, hepatoprotective, antitumor, wound‐healing, antidiabetic, and anti‐aging properties, and it activates the endogenous defense system (Karimian et al. 2019). Propolis and its antioxidative potential largely depend on the total amount of polyphenols and flavonoids. These antioxidant compounds can scavenge ROS by chelating transition metals. A meta‐analysis reported that propolis is a safe supplement that has beneficial effects on glutathione, glutathione peroxidase, and total antioxidant capacity levels. It can be considered an effective adjunctive treatment for diseases in which oxidative stress plays a role in the etiology (Nazari‐Bonab et al. 2023). Moayedi et al. (2023) reported that propolis supplementation with exercise reduced inflammation activity and increased the antioxidant defenses of women with diabetic dyslipidemia after 8 weeks. Propolis has an immunomodulatory effect by acting on different cells involved in the innate and adaptive immune response (Al‐Hariri 2019). Short‐term administration of a dry propolis extract at 400 mg/day reduced plasma levels of TNF‐α and macrophage inflammatory protein‐1β by alleviating inflammation in chronic kidney patients undergoing hemodialysis (Chermut et al. 2023). Another investigation found that supplementation with propolis led to an elevation in FOXP3 expression in T‐cell regulators and enhanced lymphocyte proliferation among individuals with HIV infection (Tasca et al. 2023). CRP, TNF‐α, and IL‐6 were found to decrease significantly after propolis supplementation (Shang et al. 2020). A meta‐analysis investigating the effect of propolis on inflammatory mediators found that propolis supplementation could reduce serum IL‐6, CRP, and TNF‐α levels (Gholami et al. 2024).

Bee bread is a source of antioxidant compounds, including phenolic substances and vitamin C. Therefore, it is capable of neutralizing free radicals (Yıkmış et al. 2024).

Apilarnil possesses antioxidant ability thanks to the 23 different phytochemical compounds it contains, such as fumaric acid, kaempferol, and quercetin (Inci et al. 2023).

### 4.2 Antimicrobial Effects

In honey, gluconic acid and hydrogen peroxide produced when glucose is oxidized by glucose oxidase in the bees' suprapharyngeal glands play a role in its antibacterial activity. Additionally, the osmotic effects of glucose, low pH, phenolic compounds, Maillard reaction products, antibiotic‐like peptides, and methylglyoxal properties also provide immunomodulatory and anti‐inflammatory effects (Nolan et al. 2019). Sugar, polyphenols, hydrogen peroxide, 1,2‐dicarbonyl, and bee defensin‐1 compounds found in honey have synergistic effects and antimicrobial properties. Honey exhibits antimicrobial activity against a range of gram‐negative and gram‐positive bacteria, including methicillin‐resistant strains like *Staphylococcus aureus* , *Shigella sonnei* , and *Helicobacter pylori* , as well as the yeast *Candida albicans* (Almasaudi 2021). A study examining the antimicrobial potential of various honey types toward *Streptococcus pneumoniae* , *Streptococcus pyogenes* , and *Staphylococcus aureus* determined that willow honey, heather honey, and buckwheat honey had antimicrobial effects against all considered pathogens (Romário‐Silva et al. 2022).

Propolis is effective against fungi, viruses, protozoa, and helminths, but each propolis has a different composition on the basis of the geographical region in which it is generated. Thus, different propolis samples do not exert the same activities (Rivera‐Yañez et al. 2021). Researchers have demonstrated that propolis is a powerful antiherpetic agent (Rocha et al. 2022). Another study found that daily propolis consumption may improve immune response and reduce inflammatory status in individuals with HIV/AIDS who are undergoing antiretroviral therapy (Conte et al. 2021). Since propolis reduces lipid peroxidation and improves the antioxidant system, it may be beneficial for individuals with HIV who experience intense inflammatory and oxidant activity (Tasca et al. 2023). In a randomized controlled study, research has shown that the use of propolis accelerated recovery in COVID‐19 patients (Silveira et al. 2021). In research examining propolis extract for uncomplicated upper respiratory tract infections, 83% of participants receiving a propolis oral spray demonstrated symptom regression after 3 days of treatment (Esposito et al. 2021).

Bee bread has been reported to have antimicrobial activity against *Salmonella enterica* and *Staphylococcus aureus* (Urcan et al. 2018). Furthermore, this bee product is effective against *Pseudomonas aeruginosa* , a bacterium used as a safety indicator to assess the risk of water intended for human consumption (Kahraman et al. 2022).

Royal jelly is known to have antimicrobial activity. It inhibits the proliferation of gram‐positive bacteria, including *Bacillus subtilis* and *Staphylococcus aureus* , as well as gram‐negative species such as *Escherichia coli* , *Pseudomonas aeruginosa* , and *Klebsiella pneumoniae* . Additionally, it shows antifungal activity against molds like *Aspergillus fumigatus* and *Aspergillus niger* and yeasts such as *Candida albicans* (Kieliszek et al. 2023).

Bee venom acts against pathogenic microorganisms such as bacteria, viruses, and fungi with various bioactive compounds, including melittin, apamin, and PLA2. However, bee venom can induce hemolysis and toxicity in cells. Because of these side effects, more studies on the use of bee venom are needed (El‐Seedi et al. 2020). In one such study, the side effects of bee venom after its use as an analgesic were evaluated, and it was stated that skin reactions such as itching may occur (Jang and Kim 2020).

### 4.3 Cardioprotective Effects

Polyphenols present in honey may benefit cardiovascular health by enhancing endothelial function, preventing platelet aggregation, and lowering inflammatory responses, LDL‐C oxidation, blood pressure, and oxidative stress (Palma‐Morales et al. 2023). A meta‐analysis demonstrated that honey can lower TC, LDL‐C, and fasting triglyceride levels, while raising HDL‐C. Another meta‐analysis concluded that honey does not affect serum lipid profiles (Gholami et al. 2022).

Propolis has the potential to counteract oxidative stress and inflammatory processes, both of which contribute to the development of cardiovascular diseases. Brazilian green and Brazilian red propolis exhibit a wider range of beneficial biological activities (Silva et al. 2021). According to a meta‐analysis, propolis intake was associated with a notable rise in HDL‐C levels and a marked reduction in triglyceride concentrations (Salehi‐Sahlabadi et al. 2020). In contrast, findings from another meta‐analysis indicated that propolis supplementation did not produce measurable changes in lipid profile parameters (Gheflati et al. 2021). A study in rats found that propolis significantly improved the cardiotoxicity caused by 5‐fluorouracil, a chemotherapeutic agent (Barary et al. 2022). Another study concluded that propolis supplementation reduced total TC/HDL‐C, triglyceride/HDL‐C, and non‐HDL‐C/HDL‐C ratios in rheumatoid arthritis patients, as well as decreasing CRP and monocyte chemoattractant protein‐1 levels (Maddahi et al. 2023). Intake of propolis was associated with marked reductions in triacylglycerol, LDL‐C, fasting blood glucose, HbA1c, insulin levels, insulin resistance, and systolic blood pressure relative to the control groups. In addition, HDL‐C concentrations showed a significant increase following propolis supplementation (Bahari et al. 2025).

In another study, royal jelly was found to possess antiatherogenic properties, improving vascular endothelial function (Fujisue et al. 2022).

### 4.4 Anticancer Effects

Honey demonstrates anticancer activity by modulating multiple cell signaling pathways through various mechanisms, including the induction of apoptotic, antiproliferative, anti‐inflammatory, and antimutagenic processes (Wang et al. 2023). Honey contains flavonoids, including kaempferol, catechin, and quercetin, as well as phenolic acids such as caffeic acid and gallic acid, which are among its most notable anticancer constituents. These products reportedly prevent the formation of cancer cells with their antioxidant, apoptotic, antiproliferative, immunomodulatory, and anti‐inflammatory effects (Waheed et al. 2019). Along with suppressing the growth of tumor cells, honey is effective against the side effects of cancer radiotherapy and chemotherapy, such as oral mucositis, skin toxicity, fatigue, nephrotoxicity, and neutropenia (Münstedt and Männle 2020). Research has validated the role of honey in alleviating cancer‐related fatigue and enhancing the quality of life in head and neck cancer patients following chemotherapy or radiotherapy (Ramasamy et al. 2019). Another study reported that honey was effective in managing oral mucositis in cancer patients undergoing chemotherapy or radiotherapy (Peng et al. 2022). Honey prevented mucositis in patients after radiochemotherapy, significantly reduced the degree of mucositis, and provided a rapid and painless recovery process (Liu et al. 2019).

Anticarcinogenic activity is mainly based on the suppressive effects of the antioxidant compounds found in bee pollen on ROS (Denisow and Denisow‐Pietrzyk 2016). Genistein contributes hypolipemic and anticarcinogenic properties to bee pollen (Rodríguez‐Pólit et al. 2023). It is also known that bee pollen has antimutagenic effects (Kaškonienė et al. 2020). Bee pollen can promote apoptosis, trigger the secretion of TNF‐α, exert cytotoxic effects, and inhibit both the initiation and progression of cancer cells (Denisow and Denisow‐Pietrzyk 2016). It has been found to have synergistic effects with chemotherapeutic agents and to reduce the harmful effects of drugs administered to breast cancer patients (Omar et al. 2016). In the last few years, interest in the use of bee pollen as a natural medicine in the treatment of prostate cancer has begun to increase (Tuoheti et al. 2020). In a study conducted on rats, it was determined that rapeseed bee pollen could improve benign prostate hyperplasia by regulating the expression of microRNAs such as rno‐miR‐184 in the prostate (Chen et al. 2020).

CAPE represents one of the principal bioactive constituents of propolis. This compound exhibits anticarcinogenic effects by modulating various cellular processes, including apoptosis, proliferation, differentiation, inflammation, and immune responses (Zullkiflee et al. 2022). Gajek et al. (2020) reported that CAPE reduced cytotoxicity, genotoxicity, and proapoptotic activity in human gastrointestinal cancer cells. Another study found that propolis caused a decrease in the rate of uncontrolled cell division and the rate and extent of both carcinogenesis and tumorigenesis (Salehi et al. 2022). Extracts of propolis have demonstrated chemosensitizing activity toward drug‐resistant human colon carcinoma cells (Frión‐Herrera et al. 2019). Braga et al. (2019) found that red propolis reduced azoxymethane‐induced oxidative stress and the total number of preneoplastic lesions in the distal colon. The effects of propolis, honey, and propolis‐honey mixtures on human dermal fibroblast cells were examined, and propolis and/or honey was found to affect wound healing, cell migration rate, cell viability, and proliferation (Ebadi and Fazeli 2021). In vitro studies reporting the anticancer effects of propolis and its active compounds have shown its activity against cell lines including mouth, stomach, cervix, colon, leukemia, stomach, skin, breast, and prostate cancers (Chiu et al. 2020). Propolis mediates the inhibition of cyclin‐dependent kinase and matrix metalloproteinase expression, TNF‐α, inducible nitric oxide synthase, cyclooxygenase‐1/2, lipoxygenase, prostaglandins, interleukin‐1β, and other inflammatory mechanisms in cancer (Altabbal et al. 2023). ARC, a prenylated derivative of p‐coumaric acid, imparts antioxidant, antimicrobial, anti‐inflammatory, antidiabetic, neuroprotective, gastroprotective, immunomodulatory, and anticancer activities to propolis. The anticancer effects of ARC are mediated through mechanisms such as promoting apoptosis, inducing cell cycle arrest, and suppressing p21‐activated kinase 1 activity (Shahinozzaman et al. 2020). Compounds present in propolis inhibit signaling pathways such as PI3k/AKT/mTOR, NFκB, JAK–STAT, TLR4, VEGF, and TGFβ, which are important for the initiation, progression, and metastasis of cancer (Forma and Bryś 2021). Hamzah et al. (2022) reported that using a propolis‐based mouthwash is a safe and effective approach to altering the severity of oral mucositis following radiotherapy in patients with nasopharyngeal carcinoma. Another investigation demonstrated that propolis can serve as a safe and suitable intervention to enhance nutritional status and quality of life in breast cancer patients undergoing chemotherapy; however, further research is required to clarify its efficacy in managing chemotherapy‐related side effects (Davoodi et al. 2022).

Melittin, the principal constituent of bee venom, demonstrates anticancer activity by disrupting the cell cycle and triggering apoptosis, while exerting minimal effects on normal physiological cells (Małek et al. 2023).

### 4.5 Neuroprotective Effects

Oxidative stress causes dysfunction in neurons (Azman and Zakaria 2019). Honey is considered a nootropic agent because of the flavonoids and phenolic acids it contains (Shaikh et al. 2023). In one experiment, administering Tualang honey to stressed rats resulted in notably reduced stress hormone levels and brain oxidative markers, alongside improvements in memory, antioxidant enzyme activity, and neuronal density (Azman et al. 2018). A separate study carried out on rats found that honey had a memory‐enhancing effect by upregulating brain‐derived neurotrophic factor genes (Mustafa et al. 2019). In rats with MetS, honey has been found to have positive effects on anxiety and memory, in addition to improving serum glucose and lipid levels (Arshad et al. 2020). Taking phenolic compounds before the pathological onset of AD has been found to protect neurons while reducing neuroinflammation, oxidative damage, and memory and cognitive deficits. Since these polyphenols are abundant in honey, honey consumption may have similar positive effects against AD (Pluta et al. 2023).

The neuroprotective capacity of propolis and its active constituents is largely attributed to its anti‐inflammatory actions. These anti‐inflammatory effects are considered pivotal in mitigating adverse outcomes associated with aneurysms, ischemic events, and traumatic brain injuries (Zulhendri et al. 2021). The CAPE and pinocembrin contained in propolis are components that are particularly emphasized, and they contribute beneficial effects of propolis in neurological and psychiatric diseases (Menezes da Silveira et al. 2021). It was found that the use of propolis for 6 weeks significantly reduced depression scores relative to the control group (Varzaghani et al. 2022).

Royal jelly supports the survival and functions of neurons by targeting negative conditions such as inflammation, oxidative stress, mitochondrial changes, and amyloid β toxicity (Ali and Kunugi 2020).

Hamamci et al. (2020) noted that apilarnil showed neuroprotective potential against brain damage in a rat model of sepsis and demonstrated new therapeutic effects against various neurological disorders. Apilarnil can exert anticholinergic, antiglaucoma, and antiepileptic effects through enzyme inhibition (Inci et al. 2023).

### 4.6 Antidiabetic and Anti‐Obesity Effects

Research has shown that honey consumption significantly reduces fasting blood glucose and alanine amino transferase levels and increases IL‐6 levels (Ahmed et al. 2023).

Although bee pollen is used as a nutraceutical in the treatment of diabetes and obesity, its mechanism of action has not been clearly explained. PAPPs were administered to mice fattened by a high‐glucose and high‐fat diet, and it was observed that PAPPs significantly improved insulin resistance, glucose tolerance, and fatty liver (Li et al. 2017).

Propolis supplementation was additionally associated with reductions in fasting plasma glucose and HbA1c levels relative to placebo (Ochoa‐Morales et al. 2023). Afsharpour et al. (2019) found that propolis may be beneficial as a dietary supplement for individuals diagnosed with T2DM, as it improves glycemic and antioxidant status and reduces insulin resistance. Iranian propolis was found to reduce postprandial blood glucose, serum insulin, insulin resistance, and inflammatory cytokines in patients with T2DM, while also preventing liver and kidney dysfunction and increasing HDL‐C concentrations (Zakerkish et al. 2019). Research involving older women indicated that propolis supplementation may contribute to reductions in body fat mass and oxidative stress (Kanazashi et al. 2023). Abbasi et al. (2023) found that propolis supplementation reduced testosterone levels, LDL‐C/HDL‐C, and hip circumference in women with polycystic ovary syndrome, as well as having positive effects on fasting insulin and insulin resistance. It has been shown that propolis may be effective in reducing waist circumference and improving the physical health and quality of life of individuals with MetS, but it does not have significant effects on other components of MetS (Sajjadi et al. 2023). In a meta‐analysis evaluating the effect of propolis on glycemic indexes and liver enzymes, propolis consumption led to a significant reduction in fasting blood glucose, insulin, glycosylated hemoglobin, homeostatic model assessment of insulin resistance, alanine transaminase, and aspartate aminotransferase (Adeli et al. 2024).

A meta‐analysis indicated that royal jelly had no significant impact on liver function or glycemic profile in the general adult population. However, studies involving longer intervention periods and non‐healthy participants showed a significant decrease in serum fasting blood glucose (Bahari et al. 2023). Another study found that royal jelly supplementation did not beneficially affect glycemia markers (Mahboobi et al. 2019). Pourfard et al. (2023) showed that short‐term royal jelly administration did not significantly improve blood sugar or the clinical course of COVID‐19 in COVID‐19 patients, but they found that it improved headache, cough, and shortness of breath in these patients.

### 4.7 Wound Healing Effects

The local application of honey was found to shorten wound healing time in cases of pressure injuries (Sankar et al. 2021). The use of honey has a positive effect on wound healing after tooth extraction in children (Mokhtari et al. 2019), and topical honey applications have been shown to be beneficial for wound healing compared to iodine‐based dressings (Zhang et al. 2021). In another study, it was shown that honey accelerates the wound healing process of palatal wounds, and it supports healing in cases of diabetic foot ulcers (Alasqah et al. 2022). Additionally, honey‐based drops accelerated the healing of corneal epithelial defects compared to standard treatment among patients with corneal ulcers (Nejabat et al. 2021).

Topical application of propolis, owing to its anti‐inflammatory and antioxidant characteristics, has been identified as a potential therapeutic approach for managing diabetic foot wounds (Mujica et al. 2019). A meta‐analysis indicated that propolis may serve as an effective therapeutic option for managing skin ulcers (Machado Velho et al. 2023). Another study concluded that propolis can be used to accelerate wound healing in patients diagnosed with sacrococcygeal pilonidal cysts (Kubat et al. 2021).

Andritoiu et al. (2021) reported that apilarnil, like propolis and honey, had a healing and re‐epithelialization effect on wounds.

### 4.8 Other Effects

A separate experiment demonstrated that administering bee pollen or a date palm pollen suspension (100 mg/kg) for 4 weeks to diabetic male rats enhanced reproductive outcomes, including testicular mass and the concentrations of testosterone, luteinizing hormone, and follicle‐stimulating hormone, along with improvements in spermatogenesis, sperm motility, and viability (Mohamed et al. 2018). Seven et al. (2020) found that oral propolis supplementation improved sperm quality and antioxidant and histological parameters in the testicular tissues of rats. Another study found that propolis reduced oxidation and inflammatory reactions caused by carbon tetrachloride exposure in testicular tissue (Hashem 2021). Abd‐Elrazek et al. (2020) found that oral intake of propolis modulated toxic effects on the male reproductive system by improving semen quality, reducing oxidation status and DNA damage, and preserving cell energy. Elashal et al. (2024) observed that in male rats, apilarnil exerted a protective role against BPA‐induced reproductive toxicity by restoring reproductive hormone balance and enhancing antioxidant activity. In another study, apilarnil showed positive effects in restoring sexual function in male kids (Kosum et al. 2022).

Bee pollen enabled body weight recovery and increased the relative weight of the gastrocnemius muscle in rats performing eccentric exercise (Ali and Kunugi 2020). Consumption of royal jelly also improved femoral bone mineral density and ameliorated decreased muscle strength in postmenopausal women (Matsushita et al. 2021). Another study showed that daily oral consumption of royal jelly for 8 weeks was effective in relieving menopause symptoms (Sharif and Darsareh 2019). Supplementation with royal jelly and coenzyme Q10 has been reported to lower lipid peroxidation, oxidative stress, and exercise‐induced muscle damage. Thus, exercise of high‐intensity performance was increased in swimmers (Ovchinnikov et al. 2022). Another study found that bee venom had a beneficial effect in the treatment of hip pain, inflammation, and mobility after inguinal hernia surgery (Othman et al. 2023). Lee et al. (2021) showed that the combined administration of bee venom and NSAIDs is suitable for the treatment of cervical disc herniation. Another study demonstrated that bee venom administration resulted in notable reductions in knee pain and enhancements in physical function (Conrad et al. 2019).

Among individuals diagnosed with NAFLD, propolis showed protective effects on hepatic steatosis and fibrosis and reduced serum CRP levels (Soleimani et al. 2021). Another investigation found that combining propolis supplementation with an 8‐week calorie‐restricted diet led to significant improvements in glucose homeostasis, hepatic fibrosis scores, and liver function among obese individuals with NAFLD (Nikbaf‐Shandiz et al. 2022). Combining aerobic–resistance training with royal jelly supplementation may serve as an effective and advisable approach to mitigate the adverse effects of Dysfunction‐Associated Steatotic Liver Disease by influencing liver enzyme activity, paraoxonase 1 levels, LDL‐C oxidation, and the lipid profile (Askari et al. 2025). Doganyigit et al. (2020) reported that in rats, apilarnil protected against lipopolysaccharide‐induced liver damage by suppressing the TLR4/HMGB‐1/NF‐κB signaling pathway.

A study reported that propolis administration was associated with improvements in quality of life among individuals with CKD or moderate renal impairment. In addition, propolis showed therapeutic effects in these patients, leading to proposals of further research (Anvarifard et al. 2023). Another study found propolis extract to be safe and well tolerated, as well as significantly reducing proteinuria in diabetic and non‐diabetic patients with CKD (Silveira et al. 2019). Another study found that 0.8 g/kg apilarnil exerted renoprotective effects in rats with sepsis by partial modulation of important markers of the local immune system reaction (Inandiklioglu et al. 2021).

Maeda et al. (2022) demonstrated that applying royal jelly significantly improved moisture content in the cheeks. A randomized controlled trial found beeswax to be more effective than breast milk in reducing postpartum nipple pain and preventing crack formation. Beeswax may serve as a protective barrier to help prevent nipple pain and fissure formation (Serhatlioglu et al. 2023). In another study, an Abexol suspension showed benefits and safety similar to those of Abexol tablets with the use of half the standard dose in patients with gastrointestinal symptoms (González et al. 2024).

Table 1 summarizes important in vitro, animal and human clinical studies to evaluate the health effects of bee products.

**TABLE 1:** Health effects of bee products: In vitro, animal and human studies.

Bee products	Pharmacological effects	Study subjects	Intervention	Outcomes	References
*In vitro studies (very low quality of evidence)*					
Bee pollen	Anticancer	MCF‐7 and L929 cell lines	Bee pollen extract was applied to cells at 5 different concentrations (2.5, 5, 10, 20, and 40 mg/mL), and cisplatin was applied at 5 different concentrations (2.5, 5, 10, 20, and 50 μmol/mL)	Bee pollen extract inhibited the growth of both cancerous and normal cell lines. Compared to cisplatin alone, cisplatin + bee pollen extract showed greater inhibition of MCF‐7 cells	Rzepecka‐Stojko et al. (2017)
Bee venom	Anticancer	MDA‐MB‐231 breast cancer cells	The cells were seeded into 96‐well plates. They were treated with different doses of bee venom (0.7, 1.5, 3, 6, and 12.5 μg/mL) or saline solution for 12, 24, 48, and 72 h	Bee venom inhibited the proliferation of MDA‐MB‐231 cells. Bee venom did not exhibit significant cytotoxic effects on peripheral blood mononuclear lymphocytes up to a dose of 12.5 μg/mL and 72 h of incubation	Zarrinnahad et al. (2018)
Bee venom	Anticancer	Cervical cancer cell line	The cells were treated with 1, 1.8, and 4 μg/mL of melittin (isolated from bee venom)	Melittin inhibited the proliferation of the cell line in a dose‐ and time‐dependent manner. Cells treated with 4 μg/mL melittin significantly lost their morphology	Shoinbayeva (2017)
*Animal studies (very low quality of evidence)*					
Honey	Anticancer	Rats (*n* = 50) (Group 0: Healthy rats Group 1: Untreated tumor‐bearing rats Group 2: Rats receiving low dose Tualang honey Group 3: Rats receiving medium dose of Tualang honey Group 4: Rats receiving high dose Tualang honey)	Group 0: No intervention Group 1: No intervention Group 2: 0.2 g/kg/day Tualang honey Group 3: 1 g/kg/day Tualang honey Group 4: 2 g/kg/day Tualang honey	When compared to group 1, lower tumor incidence was observed in groups 2, 3, and 4. Tualang honey treatment regulated hematological parameters. In the Tualang honey treatment groups, lower anti‐apoptotic protein expression and higher proapoptotic protein expression were found	Akouchekian et al. (2018)
Bee pollen	Cardioprotective	C57BL6 ApoE knockout mice (*n* = 56)	Control group (*n* = 6): SD Intervention 1 (*n* = 10): HFD Intervention 2 (*n* = 10): HFD + 0.1 g/kg EE Intervention 3 (*n* = 10): HFD + 1 g/kg EE Intervention 4 (*n* = 10): SD + 0.1 g/kg EE Intervention 5 (*n* = 10): SD + 1 g/kg EE	In intervention 3 group, adding 1 g/kg EE to a high‐fat diet was found to protect against the development of atherosclerotic plaques in the heart arteries. Diet, reduced TC levels when supported by Supplementation with EE. Additionally, EE supplementation lowered levels of oxidized LDL‐C, ADMA, ACE, and ANG II, thereby reducing oxidative stress	Samadi et al. (2017)
Bee bread	Cardioprotective	Mice (*n* = 32)	Normal group (*n* = 8): Normal diet HFD group (*n* = 8): High‐fat diet HFD + BB group (*n* = 8): High‐fat diet + 0.5 g/kg/day bee bread HFD + orlistat group: High‐fat diet + 10 mg/kg/day orlistat	Five phenolic compounds (isorhamnetin, kaempferol, apigenin, ferulic acid, caffeic acid) were detected in the bee bread. Total cholesterol and LDL‐C levels were significantly lower in the HFD + BB group compared to the HFD group. Atherogenic index was significantly lower in the HFD + BB and HFD + O groups compared to the HFD group	Pan et al. (2019)
Royal jelly	Cardioprotective	Spontaneously hypertensive rats and Wistar Kyoto rats [WKR group (*n* = 6), SHR‐control group (*n* = 6), SHR‐royal jelly group (*n* = 6)]	KHR‐royal jelly group: 1 g/kg/day royal jelly	Compared to the SHR‐control group, the SHR‐royal jelly group showed significant reductions in SKB and DKB from the 3rd week onwards. Nitric oxide levels significantly increased in the SHR‐royal jelly group	Chiu et al. (2017)
Royal jelly	Neuroprotective	Ovariectomized rats	E2 group: 0.25 mg/90 days 17β estradiol Royal jelly group: 10 mL/kg/day royal jelly	In rats with removed ovaries, memory impairment and depression decreased after royal jelly administration. The levels of myelin and protein content in the brains of the royal jelly group were higher compared to the E2 group	Al‐Sarray and Al‐Shaeli (2022)
Bee venom	Antidiabetic	Control group: Healthy mice (*n* = 5) Diabetes group: Diabetic mice without treatment (*n* = 5) Metformin group: Diabetic mice receiving metformin (*n* = 5) Bee venom group: Diabetic mice receiving bee venom (*n* = 5)	Control group: No intervention Diabetes group: No intervention Metformin group: 150 mg/kg metformin Bee venom group: 1 mg/kg bee venom	Bee venom exhibited similar properties to metformin	Jung et al. (2018)
Apilarnil	Other effects	Breeding rams	‐Group 1: ND (Normal Diet) Group 2: ND + 10 mg/kg apistimul preparation Group 3: ND + 15 mg/kg apistimul preparation Group 4: ND + 20 mg/kg apistimul preparation (Apistimul preparation: apilarnil + NaCl)	The addition of Apistimul preparation at a dose of 15 mg/kg increased sperm concentration, motility, and fertilization capacity	Yemets (2020)
Apilarnil	Other effects	Large white gilts	Control group: ND Experimental group: ND + 0.5 g/day homogenized apilarnil	Including Apilarnilin in the diet significantly reduced diene conjugates and positively affected reproductive indices	EFSA (2018)
*Human studies (high quality of evidence)*					
Honey	Anticancer	75 HNC patients	75 HNC patients were randomly assigned to honey, Taiwanese green propolis, or usual‐care groups. Data were collected weekly for 12 weeks post‐radiotherapy	Honey is an effective therapy for managing oral mucositis severity in HNC patients undergoing radiotherapy. Although Taiwanese green propolis reduces oral mucositis severity, it is less effective than honey	Jen et al. (2025)
Honey	Neuroprotective	Patients diagnosed with MND (*n* = 60)	Control group: Anti‐MND drug + 2 placebo capsules + 1 teaspoon white sugar extract Intervention group: Anti‐MND drug + 2 capsules/day (500 mg reed extract + 30 mg saffron extract/capsule) + 1 teaspoon astragalus honey	In the intervention group, cognitive test scores increased significantly, and there was an improvement in depression scores	Ashraf et al. (2023)
Honey	Antimicrobial	Medium severe COVID‐19 patients (control group *n* = 103, intervention group *n* = 107) Severe COVID‐19 patients (control group *n* = 53, intervention group *n* = 50)	Control group Standard treatment Intervention group: Standard treatment + honey (1 g/kg/day) + black cumin seeds (80 mg/kg/day)	In the intervention group, symptoms improved significantly, and mortality decreased compared to the placebo group	Omar et al. (2016)
Honey	Antimicrobial	Children undergoing extracapsular tonsillectomy four postoperative treatment groups: standard treatment alone; Marri honey (from Western Australia); Manuka honey (from Western Australia); or placebo	The intervention groups took 5 mL of honey or placebo, six times a day, for at least 7 days, in addition to usual discharge analgesia (standard treatment)	Two to three doses daily of oral honey/placebo in children post‐extracapsular tonsillectomy for 7 days, did not result in a clinical improvement in pain or recovery over a 14‐day follow‐up period	Sommerfield et al. (2025)
Honey	Other effects	Pre‐school children diagnosed with functional constipation allocated to either a control group or a treatment group	Although both groups received standard care, the treatment group also received honey suppositories	Honey suppositories offer a promising therapeutic intervention for pediatric functional constipation, with significant clinical benefits over standard care	Yu et al. (2025)
Propolis	Antidiabetic	Patients with T2DM (*n* = 66)	Placebo group: Placebo pill (3 times/day) Intervention group: Propolis pill (300 mg/3 times/day)	In the intervention group, FBG levels decreased, whereas in the placebo group, FBG levels increased. In the intervention group, total cholesterol levels increased significantly less compared to the placebo group	Alassaf et al. (2021)
Propolis	Antidiabetic	Healthy participants (*n* = 34)	Propolis capsule (1000 mg/day propolis)	BMI and body weight increased significantly. FBG and HbA1c decreased significantly	Fukuda et al. (2015)
Propolis	Antidiabetic	Individuals with T2DM (*n* = 80); 8 weeks	Placebo group: 1 tablet/day placebo tablet (containing sunflower oil, wheat germ oil, perilla oil) Intervention group: 226.8 mg/tablet/day Brazilian green propolis	No significant differences were observed between the two groups in HOMA‐IR, insulin, HbA1c, and FBG values. In the placebo group, there was a significant increase in uric acid levels, whereas in the intervention group, it remained the same as the baseline level. In the placebo group, eGFR decreased significantly, whereas in the intervention group, it remained the same as the baseline level	Mujica et al. (2017)
Propolis	Cardioprotective	Volunteer participants (*n* = 67)	Placebo group: Mixed drop (mint, fern, synthetic) 15 drops/2 times/day Intervention group: 3% propolis solution 15 drops/2 times/day	In the intervention group, HDL‐C and glutathione levels increased significantly compared to the placebo group, whereas TBARS decreased significantly	Asama et al. (2021)
Propolis	Neuroprotective	Elderly (60–79 years) Japanese individuals (*n* = 68)	Placebo group: 6 placebo capsules/day Intervention group: 6 propolis capsules/day	In the propolis group, significant improvements were observed in verbal memory and processing speed compared to the placebo	Othman et al. (2019)
Propolis	Antimicrobial	COVID‐19 patients (*n* = 124)	Control group: Standard treatment Intervention group 1: Standard treatment + 400 mg/day Brazilian green propolis Intervention group 2: Standard treatment + 800 mg/day Brazilian green propolis	Length of hospital stay decreased in the intervention groups	Al‐Hariri (2019)
Propolis	Antimicrobial	Individuals with mild upper respiratory tract infection (*n* = 122)	Intervention group: Propolis spray 2–4 puffs, 3 times/day (12–24 mg/day polyphenol)	It was observed that the intervention group recovered faster within 5 days	Soleimani et al. (2021)
Propolis	Other effects	42 patients with CKD undergoing hemodialysis	42 patients were divided into two groups: the placebo and propolis group received 400 mg of green propolis extract/day for 8 weeks	Despite not being significant, microbial evenness and observed richness increased following the propolis intervention. Two months of propolis supplementation did not reduce the plasma levels of uremic toxins or change the fecal microbiota	Fonseca et al. (2024)
Apilarnil	Cardioprotective	Adults with mild hypercholesterolemia (180–200 mg/dL) (*n* = 40)	Placebo group: 9 capsules/day of placebo capsules Intervention group: Royal jelly capsule (350 mg/capsule of royal jelly) 9 capsules/day	In the intervention group, total cholesterol and LDL‐C significantly decreased, whereas DHEA‐S levels increased significantly	Minami et al. (2016)

Abbreviations: ACE, angiotensin‐converting enzyme; ADMA, asymmetric dimethylarginine; ANG II, angiotensin II; BB, bee bread; BMI, body mass index; BNP, brain natriuretic peptide; BPE, bee pollen extract; CKD, chronic kidney disease; COVID‐19, coronavirus disease 2019; DBP, diastolic blood pressure; DHEA‐S, dehydroepiandrosterone sulphate; ECP, *E. coli* polysaccharides; EE, high polyphenol ethanol extract from bee pollen; Egfr, estimated glomerular filtration rate; FBG, fasting blood glucose; HbA1c, hemoglobin A1c; HDL‐C, high‐density lipoprotein cholesterol; HFD, high‐fat diet; HNC, head neck cancer; HOMA‐IR, homeostasis model assessment of insulin resistance; LDL‐C, low‐density lipoprotein cholesterol; LH, luteinizing hormone; MND, major neurocognitive disorder; ND, normal diet; O, orlistat; PCOS, polycystic over syndrom; SBP, systolic blood pressure; SD, standard diet; SHR, spontaneously hypertensive rats; T2DM, type 2 diabetes mellitus; TBARS, thiobarbituric acid reactive substances; TC, total cholesterol; TNF‐α, tumor necrosis factor alpha; WKR, Wistar Kyoto rats.

## 5 Conclusion and Recommendations

Bee products such as honey, pollen, propolis, bee bread, royal jelly, bee venom, beeswax, and apilarnil exhibit a wide range of biological activities, including antioxidant, anti‐inflammatory, antimicrobial, cardioprotective, anticancer, neuroprotective, antidiabetic, and wound‐healing effects. Their therapeutic potential is attributed to various bioactive compounds, such as flavonoids, polyphenols, amino acids, and fatty acids, which contribute to immune modulation and protection against oxidative stress‐related diseases. This review highlights the role of bee products in the prevention and management of conditions such as cancer, diabetes, cardiovascular and neurological disorders. However, variability in the composition of bee products and the lack of standardized preparations remain major obstacles to translating these findings into clinical practice. Therefore, well‐designed clinical and epidemiological studies are needed to confirm their efficacy, ensure their safety, and determine the optimal dosage for apitherapy.

### 5.1 Safe Levels and Toxic Effects

According to a report of the EFSA, the advised dosage of propolis is 0.7–1.3 g/day and the safe use limit is 2 g/kg/day. The daily dose intake with food supplements is 24–72 mg of propolis. The use of propolis extract is advised to be at least 250 mg/day for children and 750 mg/day for adults, 1 or 3 times a day (EFSA 2018). According to the WHO, the consumption of simple sugars should be limited to under 10% of total energy intake. It also emphasizes that it is important to reduce simple sugar intake to less than 5% of total energy for additional health benefits (WHO 2024). Simple sugar or added sugar comes from natural sugars found in honey and fruit juices (EFSA 2018). Considering that a healthy adult consumes an average of 2000 kcal/day and can meet the advised simple sugar intake with honey alone, it can be recommended that adults consume a maximum of ~3 tablespoons of honey daily as, in the view of USDA, 1 tablespoon of honey contains 16.4 g of sugar.

Some toxicity factors in honey may raise concerns about its safety. In the presence of a toxic situation, the health of the individual may be negatively affected (Bahari et al. 2023). *Clostridium botulinum*, a bacterium that may occur in both honey and bee pollen, is capable of producing eight distinct toxins and is regarded as a potential risk factor for infant botulism. WHO advises against giving honey to infants younger than 1 year (Maikanov et al. 2019). Harmful effects of HMF on human health, which can be formed by the hydrogenation of sugary substances in honey as a result of the Maillard reaction under high temperature and low pH conditions, have been detected (Li et al. 2020). According to the International Honey Commission Standards, the HMF content of all honey should be a maximum of 40 mg/kg (Bogdanov 2024). Since naphthalene has numerous toxic properties, it is a widely used chemical against all kinds of harmful insects and moths. Beekeepers keep their hives and empty honeycombs safe from moths, insects, and mold until the next year with mothballs used for preservation. Thus, naphthalene penetrates into the comb and honey and forms a residue (Murcia‐Morales et al. 2023). The limit value for the naphthalene that can be found in honey is 10 mg/kg according to European Union regulations (EC 2024; EFSA 2024).

### 5.2 Future Perspective

The lack of standardization of bee products hinders the clarity of studies. Further research employing standardized bee‐derived products is essential to gain a clearer understanding of their health effects. Moreover, variations in ethnicity add another layer of complexity to interpreting diverse findings, and certain medications may influence the effects of these products in different ways. Since no clinical studies have reached phase III to date, it is not possible to directly apply these products in clinical settings. For this reason, dose–response relationships need to be evaluated and standardization needs to be done. In future studies, examining the components of bee products in different model organisms and conducting clinical trials at various doses and durations will help reveal potential relationships, contribute to the understanding of the complex interactions between bee products and health, and help shape the direction of future research.

### 5.3 Limitations

Gathering solid evidence on the therapeutic and health‐promoting uses of honey and other bee‐derived products remains challenging. An important reason for this challenge is that the quantity, composition, and potential synergistic interactions of bioactive compounds with health‐promoting effects in bee‐derived products can differ according to the floral origin, the type of honey, the traits of the bees, and the concentration levels. It seems that studies on apitherapy are mostly animal studies and in vitro studies. Another limitation is determining the appropriate dose with expected health benefits. Additionally, the possibility of allergy should be taken into consideration when using bee products.

## Author Contributions

**Nevin Sanlier:** formal analysis, investigation, methodology, writing – original draft, and writing – review and editing. **Elif Yildiz Kaya:** investigation, writing – original draft, and writing – review and editing. **Ikbal Irem Yucel:** investigation and writing – original draft.

## Ethics Statement

The papers cited involving the use of human subjects, have been carried out in accordance with The Code of Ethics of the World Medical Association (Declaration of Helsinki) for experiments involving humans. The papers cited involving animal research have been carried out in accordance with the U.K. Animals (Scientific Procedures) Act, 1986 and associated guidelines, EU Directive 2010/63/EU for animal experiments, or the National Institutes of Health guide for the care and use of Laboratory animals (NIH Publications No. 8023, revised 1978).

## Conflicts of Interest

The authors declare no conflicts of interest.

## Data Availability

The data that support the findings of this study are available from the corresponding author upon reasonable request.
